# Clinicoradiologic Correlation in Bilateral Internuclear Ophthalmoplegia Revealing a Ruptured Pontine Abscess

**DOI:** 10.7759/cureus.98671

**Published:** 2025-12-07

**Authors:** Yassine Mebrouk, Fatima Ez-Zahra Mabrouki

**Affiliations:** 1 Department of Neurology, University Hospital Mohammed VI, Faculty of Medicine and Pharmacy, Mohammed I University, Oujda, MAR; 2 Department of Ophthalmology, University Hospital Mohammed VI, Faculty of Medicine and Pharmacy, Mohammed I University, Oujda, MAR

**Keywords:** brainstem lesion, internuclear ophthalmoplegia, magnetic resonance imaging, pontine abscess, ring-enhancing lesion

## Abstract

Internuclear ophthalmoplegia (INO) is an uncommon but well-recognized clinical sign, most frequently associated with demyelinating disorders or brainstem infarction. Its occurrence in the context of an infectious process, particularly a brainstem abscess, is exceptional. We report the case of a previously healthy 37-year-old man who presented with acute meningeal signs and binocular horizontal diplopia. Magnetic resonance imaging (MRI) revealed a ring-enhancing pontine lesion with diffusion restriction and posterior interruption of enhancement at the interface with the fourth ventricle, consistent with a ruptured brainstem abscess. The patient responded favorably to empirical intravenous antibiotic therapy and achieved complete clinical recovery. This case is noteworthy because of the unusual anatomical location of the abscess and its distinctive clinical presentation, underscoring the crucial role of imaging in early diagnosis. It further emphasizes that infectious etiologies should be considered in patients presenting with INO and shows how specific MRI features can help in distinguishing infectious from non-infectious pontine lesions.

## Introduction

Ring-enhancing brain lesions on neuroimaging give rise to a broad differential diagnosis, including neoplasms, infections, demyelinating disorders, and vascular insults [1]. Pontine abscesses are rare, particularly in immunocompetent adults, and their clinical presentation may be atypical, often mimicking other brainstem pathologies [2,3].

Internuclear ophthalmoplegia (INO), which results from a lesion of the medial longitudinal fasciculus (MLF), is traditionally associated with demyelinating diseases such as multiple sclerosis or with brainstem infarction. Infectious causes of INO remain exceptionally rare [4,5].

This case is remarkable for the exceptional pontine location of the abscess and its rare presentation with INO. It highlights the diagnostic value of brain magnetic resonance imaging (MRI) in identifying infectious brainstem lesions and underscores the need to consider infectious etiologies when INO appears in an atypical clinical context.

## Case presentation

We report the case of a previously healthy 37-year-old man with no personal or family history of Behçet's disease, autoimmune disorders, neoplasia, or immunosuppression. He developed subacute meningoencephalitic syndrome beginning with intense diffuse headache resistant to analgesics, accompanied by projectile vomiting. Within a few days, he developed persistent horizontal diplopia, more pronounced on lateral gaze. Visual acuity was preserved, and he denied any motor or sensory deficits, seizures, or focal neurological symptoms. On admission, he was febrile at 39°C (≈102.2°F) and fully conscious. Neurological examination revealed clear signs of meningeal irritation, including neck stiffness and photophobia. Oculomotor evaluation demonstrated striking bilateral INO characterized by adduction failure with contralateral abducting nystagmus, while convergence was preserved, suggesting bilateral involvement of the MLF. No other cranial nerve deficits or cerebellar signs were noted.

Contrast-enhanced cerebral computed tomography (CT) showed diffuse meningeal enhancement without focal lesions. Cerebrospinal fluid (CSF) analysis revealed 737 leukocytes/mm³ (60% neutrophils), a protein level of 0.88 g/L, and a markedly reduced glucose level of 0.24 g/L. Although Gram stain and bacterial cultures were negative, the combination of neutrophilic pleocytosis, high protein, and low glucose strongly supported bacterial meningitis. Extensive microbiological investigations, including Ziehl-Neelsen staining, GeneXpert *Mycobacterium tuberculosis*/rifampicin (MTB/RIF) testing, polymerase chain reaction (PCR) assays for herpesviruses and *Toxoplasma gondii*, human immunodeficiency virus (HIV) serology, *Treponema pallidum *hemagglutination assay (TPHA), Venereal Disease Research Laboratory (VDRL) test, and QuantiFERON-TB Gold, were all negative. Blood tests demonstrated significant neutrophilic leukocytosis (16,270/mm³) and an elevated C-reactive protein level of 90 mg/L (Table 1).

**Table 1:** Laboratory findings CSF: cerebrospinal fluid, MTB/RIF: *Mycobacterium tuberculosis*/rifampicin, PCR: polymerase chain reaction, HIV: human immunodeficiency virus, TPHA: *Treponema pallidum* hemagglutination assay, VDRL: Venereal Disease Research Laboratory, TB: tuberculosis, CRP: C-reactive protein.

Test	Patient value	Reference range
CSF cytology	737/mm³ (60% neutrophils)	<5/mm³
CSF protein	0.88 g/L	0.15-0.45 g/L
CSF glucose	0.24 g/L	0.45-0.75 g/L
CSF Gram stain	Negative	Negative
CSF bacterial culture	Negative	Negative
GeneXpert for *Mycobacterium tuberculosis*/rifampicin (MTB/RIF)	Negative	Negative
PCR for herpesviruses	Negative	Negative
PCR for *Toxoplasma gondii*	Negative	Negative
HIV serology	Negative	Negative
TPHA serology	Negative	Negative
VDRL serology	Negative	Negative
QuantiFERON-TB Gold	Negative	Negative
White blood cell count	16,270 /mm³	4,000-10,000 /mm³
CRP	90 mg/L	<5 mg/L

**Figure 1:**
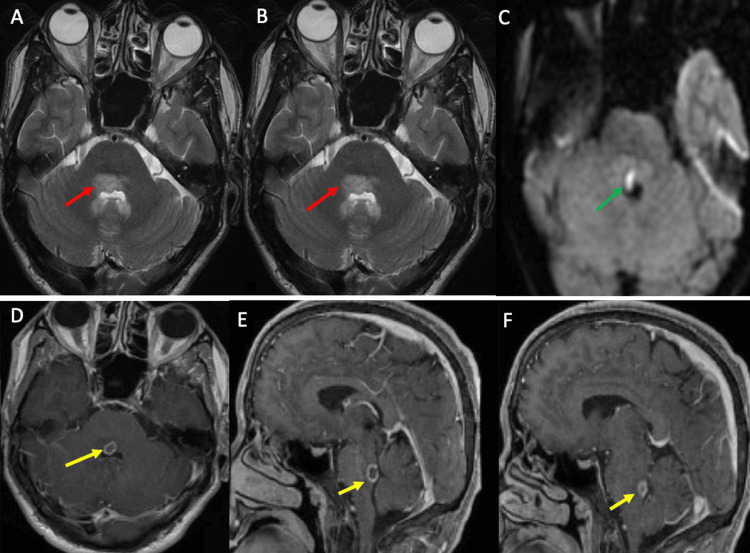
Brain MRI findings (A, B) Axial and sagittal T2WI show a 9 × 8 mm hyperintense pontine lesion located anterior to the fourth ventricle (red arrows). (C) DWI shows marked diffusion restriction within the lesion (green arrow). (D-F) Post-contrast T1WI demonstrate peripheral ring enhancement with posterior interruption at the ventricular interface (yellow arrows). MRI: magnetic resonance imaging; DWI: diffusion-weighted imaging; T1WI/T2WI: T1-/T2-weighted imaging.

Brain MRI, performed within 48 hours, identified a 9 × 8 mm pontine lesion located anterior to the fourth ventricle. The lesion was hyperintense on T2-weighted sequences and exhibited restricted diffusion on diffusion-weighted imaging (DWI), with a low apparent diffusion coefficient (ADC). Post-contrast T1-weighted sequences showed peripheral ring enhancement that was posteriorly interrupted at the interface with the fourth ventricle (Figure 1). These findings were strongly suggestive of a pyogenic brainstem abscess with secondary rupture into the ventricular system.

A complete systemic evaluation, including transthoracic echocardiography and thoracoabdominopelvic CT, did not identify any extracranial infectious source or occult malignancy.

**Figure 2:**
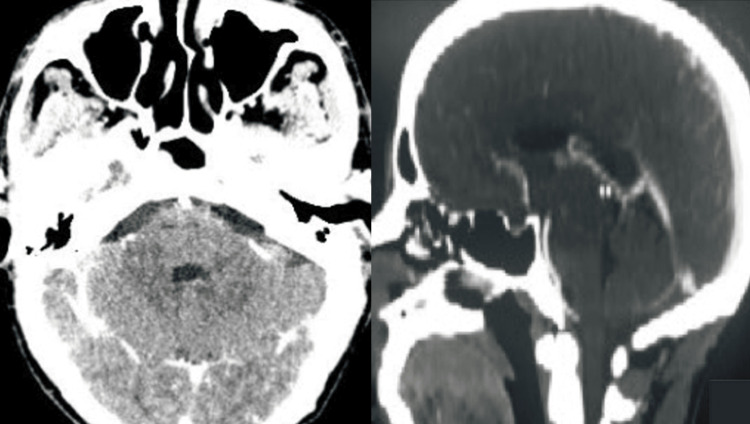
Follow-up neuroimaging findings Post-treatment axial cerebral CT images show a marked reduction in the size of the pontine lesion. The previously observed ring enhancement has resolved, consistent with a favorable therapeutic response. CT: computed tomography.

Despite the absence of an identified pathogen in the CSF, the combination of classical clinical signs of meningeal involvement, MRI features, and CSF abnormalities provided strong evidence for an infectious etiology and guided therapeutic management. The patient was promptly started on empirical intravenous broad-spectrum antibiotic therapy consisting of ceftriaxone (2 g every 12 hours), vancomycin (15-20 mg/kg every 12 hours), and ampicillin (2 g every four hours), which was continued for six weeks. Ampicillin was specifically included to target *Listeria monocytogenes*, despite its rarity as a cause of brain abscess in immunocompetent individuals. This decision was supported by two key factors: the patient’s MRI showed a ring-enhancing lesion consistent with those reported in *L. monocytogenes* abscesses, and, based on our institutional experience, *Listeria* is isolated in approximately 15% of meningitis cases treated in our department. His clinical status progressively improved, and the horizontal diplopia gradually resolved over the following two weeks. A follow-up cerebral CT scan performed three weeks later demonstrated a marked reduction in the size of the pontine lesion, with complete resolution of contrast enhancement (Figure 2). The patient completed a full six-week course of intravenous antibiotic therapy and was discharged without neurological sequelae.

## Discussion

The occurrence of a brainstem abscess, particularly within the pons, is rare, and its presentation as bilateral INO is even more exceptional. The MLF, a compact white matter tract in the dorsal pontine tegmentum, plays a critical role in coordinating conjugate horizontal eye movements. Bilateral lesions of the MLF result in INO with preserved convergence, a highly localized yet non-specific clinical sign [4,5].

In young individuals, INO is most commonly linked to demyelinating disorders such as multiple sclerosis. Vascular causes, including small brainstem infarcts, are more common in older patients. Infectious etiologies, such as bacterial abscesses, are rare but important differential diagnoses, especially in the context of systemic signs or CSF abnormalities [4,5].

The imaging findings in our patient were characteristic of a brain abscess, demonstrating the classic ring-enhancing pattern, central necrosis, and restricted diffusion on DWI. However, as emphasized by Schwartz et al. in their retrospective analysis of 221 ring-enhancing intracerebral lesions, this radiologic pattern is not pathognomonic [1]. Glioblastomas and metastases accounted for 40% and 30% of the cases, respectively, whereas only 8% were abscesses and 6% were demyelinating or granulomatous lesions [1]. These data highlight the importance of integrating clinical presentation, laboratory results, and imaging features to refine differential diagnosis. In our case, classic CSF findings of neutrophilic meningitis, along with the patient’s marked clinical and radiological improvement with antibiotic therapy, strongly support the diagnosis, helping distinguish pontine abscess from neoplastic, demyelinating, or vascular pathologies.

The detection of pontine abscess suggests a deeper pathogenic interplay. One possibility is that unrecognized primary bacteremia seeded the pons via small perforating arteries, leading to abscess formation with secondary rupture into the fourth ventricle and meningeal irritation [3]. Alternatively, primary meningitis could have extended to the brainstem parenchyma, although centripetal spread was less common [3]. In our case, the posterior interruption of ring enhancement in continuity with the fourth ventricle supports the first hypothesis. However, this interpretation is somewhat challenged by the negative results of the infectious workup for an extracerebral source.

Infectious lesions in the brainstem are diagnostically challenging, not only because of their rarity but also because of the protean clinical manifestations they may produce. Diplopia, vertigo, ataxia, or altered consciousness may all be initial presentations [2,3]. Hence, recognition of unusual clinical signs such as bilateral INO should prompt immediate neuroimaging. Moreover, the absence of immunosuppression in our patient emphasizes that serious central nervous system infections can occur even in immunocompetent hosts [6].

From a therapeutic perspective, early diagnosis and aggressive antibiotic treatment are important. Surgical drainage, often considered in cortical abscesses, is less feasible in the brainstem because of the high procedural risks. Fortunately, our patient responded well to medical treatment, reinforcing the importance of conservative management when clinical and radiological responses are favorable.

This case report has several limitations. First, the absence of microbiological confirmation represents a major limitation, as the causative pathogen could not be identified. Second, the post-treatment follow-up was performed using CT imaging only, without MRI, which may have limited the assessment of the full resolution of the lesion. Despite these limitations, the diagnosis and management were guided by clinical, radiological, and therapeutic response findings.

## Conclusions

This case presents a unique association between bilateral INO and a ruptured pontine abscess mimicking a brainstem demyelinating or neoplastic process. It highlights the key diagnostic contribution of brain MRI, particularly diffusion restriction and incomplete ring enhancement, in distinguishing infectious from non-infectious brainstem lesions. Clinicians should consider infectious etiologies even in immunocompetent patients, and integrate neurological findings, cerebrospinal fluid analysis, and high-resolution imaging when assessing atypical brainstem lesions.
